# Synergistic remediation of Pb contamination in rice field soil with FeMg-LDH@Bentonite and compost: impacts on Pb bioavailability and soil environment

**DOI:** 10.3389/fmicb.2026.1756444

**Published:** 2026-02-06

**Authors:** Xian Guan, Jing Bai, Xing Zhong Yuan, Jian Wei Guo, Xiaowen Liu

**Affiliations:** 1 College of Civil and Environmental Engineering, Hunan University of Science and Engineering, Yongzhou, China; 2 College of Environmental Science and Engineering, Hunan University, Changsha, China; 3 College of Agronomy and Life Sciences, Yunnan Urban Agricultural Engineering and Technological Research Center, Kunming University, Kunming, China; 4 College of Chemistry and Bioengineering, Hunan University of Science and Engineering, Yongzhou, China

**Keywords:** *in situ* remediation, layered double hydroxides (LDHs), paddy soil, Pb contamination, soil microbial communities

## Abstract

**Introduction:**

Lead (Pb) contamination in paddy soils can degrade soil quality, increase plant Pb uptake, and disrupt soil microbial communities. This study evaluated integrated remediation using FeMg-LDH@Bentonite (FMLB) and compost, focusing on Pb bioavailability, plant uptake, and microbial community structure.

**Methods:**

A pot-based remediation experiment was conducted using Pb-contaminated paddy soil. FMLB and compost were applied at different mixing ratios. Pb bioavailability and soil Pb levels were assessed alongside plant uptake indicators. Soil microbial community structure and alpha-diversity were characterized (e.g., by 16S rRNA gene amplicon sequencing), and soil enzyme activities were measured to reflect soil biological functioning.

**Results:**

Pb contamination significantly altered soil properties, reduced soil quality, and impacted microbial diversity. Amendment application reduced Pb bioavailability across treatments, with the greatest reduction observed for the combined treatment of FMLB:compost = 3:7. This optimal combination not only decreased Pb concentrations and plant uptake potential, but also improved microbial indicators: bacterial community composition and α-diversity metrics shifted toward the original soil (OS) reference under identical pot conditions, and enzyme activities were enhanced.

**Discussion:**

Overall, combined application of FMLB and compost represents an environmentally sound and potentially cost-effective strategy for remediating Pb-contaminated paddy soils while improving soil fertility and microbial function. Importantly, microbiome responses and selected enzyme endpoints are interpreted as indicators associated with Pb stabilization and improved soil condition, rather than direct evidence of microbially mediated Pb transformation.

## 1 Introduction

This research offers important perspectives on the effectiveness of organic-inorganic amendments in mitigating heavy metal contamination and improving soil health, supporting the creation of efficient, environmentally responsible, and *in situ* solutions for remediating agricultural soils (Mai et al., 2025). Lead (Pb) can enter agricultural systems through different human-driven actions, such as extraction of minerals, metal processing, industrial operations, and the burning of fossil fuels. Once introduced into the soil, Pb tends to accumulate over time and is often difficult to remove through natural attenuation processes. The movement of Pb from polluted land into crops, particularly staple grains like rice, presents a significant risk to both food safety and public wellbeing (Bhoi et al., 2025). Elevated Pb concentrations in agricultural soils have been reported to exceed environmental Pb standards in various regions of China, with some studies indicating that up to 15-30% of agricultural lands in certain areas (Yang et al., 2020). Chronic exposure to Pb, even at relatively low levels, can lead to severe health outcomes. Adults may experience cardiovascular and renal impairments, while children are particularly vulnerable, potentially suffering from neurocognitive deficits, reduced intelligence, attention disorders, and learning disabilities (Trachtman et al., 1984).

Traditional remediation efforts, which often rely on singular technologies such as soil acidification, deep tillage, or irrigation management, have shown limited effectiveness in permanently reducing Pb bioavailability and risks. These conventional approaches tend to be costly, time-consuming, and may lead to secondary pollution. Therefore, recent studies have focused on creating integrated, economical, and eco-friendly *in situ* remediation approaches that can both lower Pb bioavailability and enhance soil health (Paniagua López, 2024; Hamid et al., 2020). Recent comprehensive reviews have highlighted advances in soil amendments for heavy metal–contaminated soils, including the mechanisms and impacts of both natural and synthetic amendments on reducing heavy metal hazards and improving soil properties (Nie et al., 2024). Among the new soil remediation techniques, the application of materials like layered double hydroxides (LDHs), bentonite, and compost has demonstrated encouraging outcomes (Beesley et al., 2010). For example, layered double hydroxide–based composites have been demonstrated to strongly immobilize Pb and other cations in contaminated soils, significantly reducing their bioavailability under controlled conditions (Zhang et al., 2025). These amendments can immobilize Pb through various mechanisms, including adsorption, complexation, and precipitation, thereby reducing Pb mobility and bioavailability. For instance, the incorporation of specific LDH materials has demonstrated the ability to bind Pb ions, improve soil physicochemical properties, and alter soil microbial communities, collectively contributing to a stable and less bioavailable Pb fraction (Nguyen et al., 2024). Nevertheless, LDH-induced alkalization may suppress soil enzyme activity and alter microbial assemblages.

In addition to inorganic amendments, organic amendments such as compost are receiving increasing attention. Compost not only supplies essential nutrients and enhances soil structure but also increases soil organic matter content, which can complex with Pb and further reduce its bioavailability. Such organic-inorganic amendment synergisms have shown promise in creating sustainable remediation solutions (Lwin et al., 2018; Su et al., 2024; Gul et al., 2015). For example, integrating FeMg-LDH or bentonite with compost can create a multi-faceted remediation strategy, optimizing Pb immobilization while improving soil fertility and microbial activity, ultimately fostering plant growth and reducing Pb accumulation in edible tissues. From a microbiology perspective, Pb contamination can restructure rhizosphere microbial communities and impair key soil functions, and microbiome shifts can serve as sensitive indicators of remediation outcomes. Therefore, beyond Pb fractionation and plant uptake, we used 16S rRNA-based community profiling and selected enzyme endpoints to evaluate how organic–inorganic amendments reshape bacterial community structure under flooded paddy conditions. This allows us to discuss remediation not only as a geochemical stabilization process but also as a microbiome-associated recovery process under controlled pot conditions.

Accordingly, this study addresses two intertwined hypotheses: (i) co-application of FMLB and compost will immobilize Pb more efficiently than single-component treatments by uniting physicochemical adsorption with organic complexation, and (ii) the organic fraction will offset any adverse alkaline shift induced by FMLB, thereby aiding recovery of enzyme activity and bacterial diversity. To test these hypotheses, we quantified Pb fractionation, plant uptake, soil physicochemical indices and 16S rRNA-based bacterial community structure under three FMLB/compost ratios. The findings would refine amendment design for sustainable, *in situ* remediation of Pb-contaminated paddy fields.

## 2 Materials and methods

### 2.1 Preparation of FMLB composite and Pb-contaminated soil samples

The composites were prepared following the method described in our previously published study (Guan et al., 2022). Soil without contamination was collected from Shangmujing Village in Jielvqiao Town, Yongzhou City, China (26°N, 111°E), and brought to the lab for analysis. After being left to dry for a month, the soil was crushed and passed through a 10-mesh sieve. Lead nitrate (Pb (NO_3_)_2_) was incorporated into the dried soil at a concentration of 480 mg/kg to introduce Pb. The mixture was subsequently kept at 25 °C for 8 weeks to ensure the stability of the Pb-contaminated soil. The total Pb concentration was determined by ICP-MS (ICP-MS-7900, Agilent, USA), yielding a value of 534.62 mg/kg. The Pb spiking level (480 mg/kg as Pb (NO_3_)_2_; final total Pb 534.62 mg/kg) was selected to represent a severe contamination scenario. This level is substantially higher than the risk-based thresholds for Pb in Chinese agricultural soils (GB 15618-2018; thresholds are pH-dependent; e.g., 120 mg/kg for 6.5 < pH ≤ 7.5), allowing detectable geochemical and microbiome responses under pot conditions. Therefore, the results should be interpreted as remediation performance under a high-exposure (worst-case) scenario rather than as representative of typical background farmland soils. The original uncontaminated soil (OS) served as the negative control. OS was collected from the same site and processed using the same air-drying, sieving, pot setup, rice cultivation, water (paddy/flooded) regime, incubation/growth duration, and sampling procedures as the Pb-contaminated soils. The only differences were that OS received neither Pb(NO3)_2_ spiking nor amendment addition.

### 2.2 Experimental procedures and analytical techniques

The effects of the treatments on Pb mobility and the soil's physical and chemical characteristics were evaluated. Additionally, the impact of remediation methods was assessed by measuring plant Pb levels, microbial composition, and enzyme activities in the treated soil. Amendments were introduced to the soil at 3% of its dry mass, with varying ratios of FMLB to compost (3:7, 1:1, 7:3). The amendment rate (3%, w/w) was selected based on our previous pot-scale studies with related composite amendments, where this dosage provided stable and measurable responses under controlled conditions. Accordingly, the present study is intended as a proof-of-concept pot experiment, and field application would require further dosage optimization and validation under realistic agronomic management. The original uncontaminated soil (OS) was run in parallel under the same pot conditions (rice cultivation, flooded water regime, experimental duration, and sampling procedures) as all Pb-spiked treatments, but without Pb spiking and without amendment addition. Each treatment (including OS and CK) was replicated in 3 pots, with each pot containing 3 plants. Amendments were incorporated into the contaminated soil, which was then transferred into pots with a diameter of 27 cm and a height of 28 cm. The soil was kept saturated with water, and rice (Youxiangyoulongsimiao) was grown during the 120 day remediation period.

#### 2.2.1 Soil chemical and physical characteristics

After the remediation treatment, soil samples were taken from each pot, dried in the air, and ground to a 2 mm particle size. Subsequently, the water-soluble organic carbon (WSOC), organic matter (OM), and pH were measured. The pH was measured using a pH meter (FE28-Standard, Mettler Toledo) with a soil-to-water ratio of 1: 2.5 for the suspension (Zhao et al., 2018). WSOC was measured with an automated TOC analyzer (Shimadzu L-series, TOC-CHP, Japan) using a 1:10 soil-to-water ratio and ultrapure water. OM content was determined by drying the samples at 105 °C until constant weight was achieved, followed by combustion in a muffle furnace at 550 °C for 4 h (Lu et al., 2018).

#### 2.2.2 Extraction of metal fractions

The heavy metals were separated into four distinct fractions using the BCR (Community Bureau of Reference) extraction technique, with specific procedures outlined in Supplementary Table 1 (Xiong et al., 2018). This method was used to analyze the role of the amendments in altering the biological availability of Pb.

#### 2.2.3 Measurement of Pb concentration in plant samples

After harvesting, the rice plants were thoroughly rinsed with running water, followed by deionized water, to remove any adhering soil particles and contaminants. Subsequently, the roots, shoots, leaves, and grains were separated and subjected to drying at 60 °C until they reached a constant weight. The plant sample (0.50 g) was treated by a wet digestion method using 6 mL of HNO_3_ + 1.5 mL of HClO_4_, which were determined with ICP-MS (ICP-MS-7900, Agilent, American).

#### 2.2.4 Amplification and sequencing of 16S rRNA genes

A total of 21 soil samples were collected and processed for DNA extraction and 16S rRNA gene sequencing. Soil samples of total DNA (about 2.5 g) were extracted with the FastDNA^®^ SPIN DNA Kit for soil (MP Biomedicals, U.S.). The DNA extract was checked on 1 % agarose gel, and DNA concentrations and purities were measured using a NanoDrop 2000 UV-vis spectrophotometer (Thermo Scientific, Wilmington, USA). The highly variable region V3-V4 of the bacterial 16S rRNA gene was amplified on an ABI GeneAmp^®^ 9700 PCR thermal cycler (ABI, CA, USA) using primer pairs 338F 5′-ACTCCTACGGGAGGCAGCAG3′) and 806R 5′-GGACTACHVGGGTWTCTAAT3′). PCR reactions were performed in triplicate. PCR products were extracted from 2 % agarose gels and quantified using Quantus™ fluorometer (Promega, USA) using the AxyPrep DNA Gel Extraction Kit (Axygen Biosciences, Union City, CA, USA) and purified according to the manufacturer's instructions. The purified amplicons were pooled in an equimolar and paired-end sequencing was performed on the Illumina MiSeq PE300 platform (Illumina, San Diego, USA) following the protocol standard of Majorbio Bio-Pharm Technology Co. Ltd. (Shanghai, China). Raw 16S rRNA gene sequence reads were demultiplexed, quality-filtered using fastp v0.20.0, and merged using FLASH v1.2.7 (Magoč and Salzberg, 2011). OTUs were clustered at 97% sequence similarity using UPARSE v7.1, and chimeric sequences were identified and removed (Stackebrandt and Goebel, 1994; Edgar, 2013). The RDP classifier version 2.2 was used to analyze the classification of each OTU representative sequence against a 16S rRNA database (e.g., Silva v138) with a confidence threshold of 0.7 (Wang et al., 2007). Alpha diversity indices, including Chao1, ACE, and Shannon, were calculated to evaluate bacterial richness and evenness within each treatment group. Beta diversity was assessed using Bray–Curtis dissimilarity and visualized by non-metric multidimensional scaling (NMDS) to explore differences in bacterial community composition among treatments. Statistical significance of community structure differences was tested using PERMANOVA. All diversity analyses were performed on Majorbio's online platform (https://cloud.majorbio.com/) using default parameters unless otherwise specified. We note as a methodological limitation that OTU clustering at 97% similarity provides lower taxonomic resolution than ASV-based pipelines (e.g., DADA2 denoising), which are increasingly the standard in microbiome studies; however, to maintain consistency with our original workflow and because raw-data reprocessing was beyond the scope of this revision, we retained the OTU-based approach and interpret community-level patterns cautiously, particularly for fine-scale taxonomic inferences (Chen et al., 2018).

#### 2.2.5 Enzyme activity analysis

Enzyme activities of urease and catalase were assessed by analyzing pooled, air-dried samples collected in triplicate on day 120 (Li et al., 2008; Cang et al., 2009; Huang et al., 2015b). Urease activity was quantified using a colorimetric method with toluene and a 10% urea solution, while catalase activity was assessed through titration with potassium permanganate. The effect of combining FMLB and compost on enzyme activity was assessed by performing a one-way analysis of variance (ANOVA). A *p*-value of less than 0.05 was considered statistically significant. Statistical analyses were carried out with SPSS software, version 22 (Chicago, IL).

### 2.3 Statistical analysis

All statistical analyses were performed using SPSS 22.0 (Chicago, IL, USA) unless otherwise stated. Differences among treatments were assessed by one-way analysis of variance (ANOVA), and mean separation was conducted using Tukey's HSD test. Statistical significance was set at *p* < 0.05. The original uncontaminated soil (OS) was maintained under identical pot conditions and was included in statistical comparisons only for variables measured in OS. For variables determined only for Pb-spiked soils, statistical comparisons were conducted among CK and A–E only. For microbial community analysis, Bray–Curtis dissimilarity was visualized by non-metric multidimensional scaling (NMDS), and differences in community structure among groups were tested using PERMANOVA.

## 3 Results

### 3.1 Characteristics of soil and compost

The physical and chemical properties of soil and compost are shown in Table 1.

**Table 1:** Physical and chemical properties of original uncontaminated soils and compost.

**Sample**	**pH**	**Cation exchange capacity, CEC (cmol kg^−1^)**	**Electrical Conductivity (mS m^−1^)**	**Total Nitrogen (g kg^−1^)**	**Total Phosphorus (g kg^−1^)**	**Cd (mg kg^−1^)**	**Zn (mg kg^−1^)**	**Pb (mg kg^−1^)**
Soil	7.86	24.7	22.0	3.49	0.52	0.548	88.19	40.34
Compost	7.03	38.5	594	4.3	-	ND	280.6	15.12

“–” indicates that the parameter was not measured; “ND” indicates that the parameter was measured but not detected.

### 3.2 Impact of amendments on soil physicochemical properties

**Figure 1:**
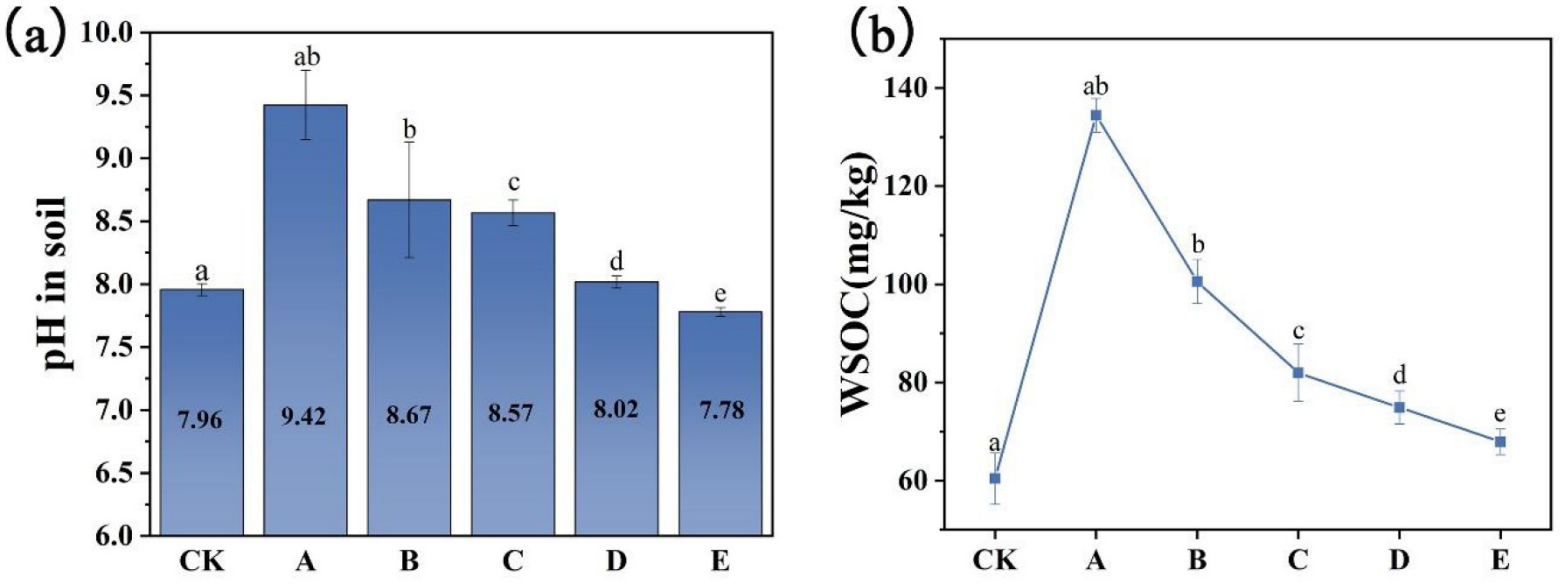
The pH values of soil **(a)** and water-soluble organic carbon levels **(b)** after applying different amendments. Error bars represent the standard deviation from three measurements. Different letters denote statistically significant differences between groups at *p* < 0.05. The treatments were as follows: CK (control), A (FMLB), B (FMLB + compost at a 7:3 ratio), C (FMLB + compost at a 1:1 ratio), D (FMLB + compost at a 3:7 ratio), and E (compost).

Amendments lead to changes in the pH levels of the soil. As illustrated in Figure 1a, a slight addition of FMLB raises the pH from 7.96 to 9.42, owing to its alkaline properties (Guan et al., 2022). Interestingly, the pH value decreased after introducing compost, with the decrease being inversely proportional to the compost concentration. The breakdown of organic matter, like compost, releases acidic compounds into the soil. Compost application also enriches the rhizosphere with humic acid, further contributing to the reduction in soil pH. Moreover, compost contains WSOC that microbes can readily break down through acidic secretions, which also aids in lowering the soil pH. When the ratio of FMLB to compost is 3:7, the pH value is 8.02, which is very similar to that of pure FMLB and compost. This occurrence is probably a result of the compost's neutral pH (Katoh et al., 2015). In acidic environments, heavy metals are more readily available to plants and exhibit increased mobility. In alkaline environments, the solubility of heavy metals decreases, possibly due to hydrolysis and electrostatic interactions (He et al., 2017).

As shown in Figure 1b, the level of WSOC in soils treated with FMLB and compost was notably greater than in the control group. Upon the addition of FMLB, the WSOC level increased by 2.23 times compared to the control, likely due to the pH change. Surprisingly, increasing the amount of compost resulted in a reduction in WSOC levels. While the addition of FMLB enhanced both pH and WSOC in comparison to the control group, incorporating compost led to a decrease in WSOC, which aligns with findings from previous research (Bolan et al., 2003). The increase in WSOC levels could play a role in lowering the long-term availability of heavy metals. WSOC shows a positive correlation with compost as a result of microorganisms decomposing readily biodegradable materials. Previous studies suggest that the rise in WSOC levels may result from the breakdown of organic compounds or the activity of microbial communities in the soil (Katoh et al., 2015).

OM is crucial for supplying nutrients that support the growth and development of microorganisms. The introduction of FMLB has a limited effect on the organic matter content in the soil, and its presence is inversely related to the quantity of compost added (Supplementary Figure 1). Compost contains a high concentration of organic matter, which helps boost its content in the soil (Liu et al., 2020).

### 3.3 BCR extraction results

**Figure 2:**
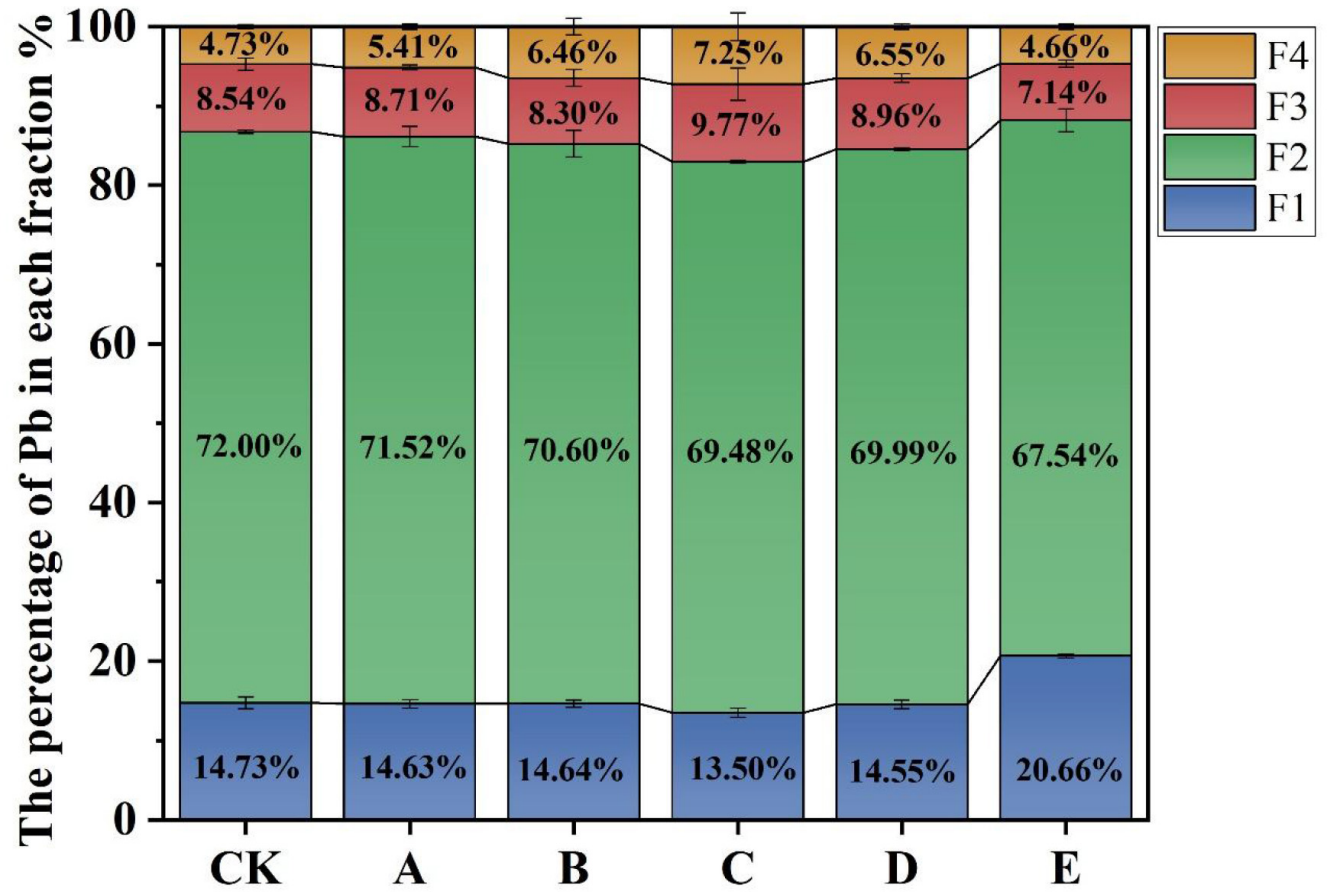
Pb fractions analyzed through the BCR sequential extraction method. Treatments: CK (control), A (FMLB), B (FMLB + compost 7:3), C (FMLB + compost 1:1), D (FMLB + compost 3:7), E (compost). F1: acid-soluble fraction; F2: reducible fraction; F3: oxidizable fraction; F4: residual fraction. Error bars represent mean ± SD (n = 3). Because sequential extraction has inherent analytical uncertainty and the replicate number is limited, fraction-wise inferential statistics were not used to support strong claims; therefore, the BCR results are interpreted primarily as compositional trends rather than definitive transformations.

To examine how heavy metals are distributed after being immobilized, the BCR sequential extraction technique was employed. The fractions that are soluble and reducible are more readily available for absorption by plants, whereas the oxidizable and residual fractions are less accessible and have a reduced potential for bioavailability. As depicted in Figure 2, incorporating amendments resulted in a decrease in both acid-soluble and reducible fractions, while increasing the oxidizable and residual fractions. This shift suggests a redistribution of Pb from more labile fractions (F1–F2) toward less labile fractions (F3–F4) after amendment application. The observed redistribution of Pb fractions suggests enhanced Pb immobilization, potentially related to sorption/complexation and precipitation processes under altered soil chemical conditions. These patterns are consistent with Pb immobilization via adsorption/complexation and potentially precipitation processes reported in previous studies (Qian et al., 2022).

In untreated plots, Pb fractions in the soil are distributed as follows: reducible (F2) > acid-soluble (F1) > oxidizable (F3) > residual (F4). The application of various composite remediation agents changed the Pb speciation in the contaminated soil. Notably, Group C showed slight shifts in Pb fractionation after 120 days of treatment. In comparison to the control (CK), F1 and F2 decreased by 1.23 % and 2.52 %, respectively, whereas F3 and F4 saw increases of 1.23 % and 2.52 %, respectively. Given the inherent uncertainty of sequential extraction, small percentage shifts should be interpreted cautiously and are discussed primarily as compositional trends. The FMLB exhibited a peak Pb adsorption capacity of approximately 1397.62 mg/kg in aqueous solutions (Guan et al., 2022). This suggests that FMLB likely immobilized the free-form Pb through surface complexation, ion exchange, and chemical precipitation. In Group E, compared with CK, F1 increased by 5.93 %, while F2, F3, and F4 also increased. Interestingly, the incorporation of pure compost did not lead to a significant reduction in Pb in its available state. The FMLB played a key role in Pb immobilization, while the compost contributed essential nutrients during decomposition, improving soil fertility and structure (Hamid et al., 2019). Other environmental factors and soil texture might also influence the adsorption of Pb (Huang et al., 2015a).

After the application of FMLB, alkaline species dissolved, leading to an increase in soil pH. This higher pH may reduce Pb mobility in the soil by favoring sorption/complexation and potentially precipitation, contributing to a redistribution of Pb toward less labile BCR fractions.

### 3.4 Plant Pb results

**Figure 3:**
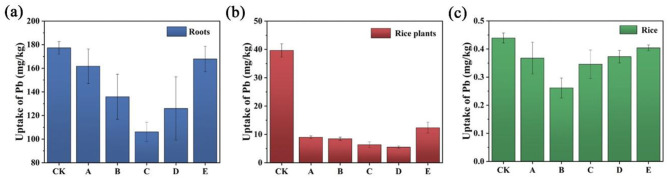
Pb accumulation in **(a)** rice roots, **(b)** rice plants (aboveground tissues), and **(c)** rice from pot experiments. Treatments: CK (control), A (FMLB), B (FMLB + compost 7:3), C (FMLB + compost 1:1), D (FMLB + compost 3:7), E (compost).

Figure 3 shows the Pb uptake by rice plants following treatment with different amendments. The Pb level in the rice roots reduced from 177.36 mg/kg to 161.82 mg/kg after applying FMLB as the remediation agent. This reduction suggests that FMLB effectively adsorbs Pb through surface complexation, ion exchange, and chemical precipitation mechanisms. Introducing compost in combination with FMLB resulted in further decreases in Pb concentration in the roots, notably reducing to 135.85 mg/kg for the 30% compost mixture. Increasing the compost proportion to 50% and 70% resulted in additional reductions to 106.12 mg/kg and 125.92 mg/kg, correspondingly. However, compost alone (Group E) as the remediation agent did not result in a significant decrease in Pb levels in the rice roots, with the concentration remaining at 167.95 mg/kg. This suggests that compost alone is not effective in reducing the mobility of Pb from the soil to the plants. These findings indicate that while FMLB alone effectively reduces Pb in plant roots, incorporating compost enhances the Pb immobilization effect and improves soil conditions. Compost also enhances rhizobacterial proliferation, soil aggregation, water retention, and pH levels, collectively improving the plant's nutrient uptake ability while mitigating heavy metal absorption (Lwin et al., 2018; Zafar-ul-Hye et al., 2020). The varying impacts of different FMLB-to-compost ratios on Pb fixation in soil highlight the importance of optimizing amendment proportions for better Pb immobilization and minimizing its transfer to plants. As shown in Figure 3b, Pb concentrations in rice plants (aboveground tissues) decreased markedly after amendment application. Compared with CK (39.63 mg/kg), Pb concentrations were reduced to 9.00, 8.45, 6.37, 5.47, and 12.37 mg/kg in Groups A, B, C, D, and E, respectively, with the lowest value observed in Group D (FMLB + compost, 3:7).

As shown in Figure 3c, the Pb content in rice (edible grain) of the control group (CK) was 0.44 mg/kg. Application of FMLB-containing amendments lowered grain Pb concentrations to as low as 0.26 mg/kg (Group B). The incorporation of compost with FMLB resulted in further reductions of Pb content to 0.37 mg/kg, 0.35 mg/kg, and 0.37 mg/kg in Groups A, C, and D, respectively. In Group E (compost alone), the Pb content decreased to 0.40 mg/kg, slightly below that of the CK group. All treatments helped reduce the Pb concentration in rice (edible grain), with Group B achieving a 41 % decrease in Pb content relative to the control group (CK). The inclusion of compost enhanced the energy supply for rhizobacteria and facilitated oxygen transfer within the soil, significantly contributing to the immobilization of metallic ions (Zafar-ul-Hye et al., 2020). And the attachment of heavy metals to exchange sites diminishes their availability for uptake by plants (Song and Greenway, 2004). The decrease in available Pb in the soil and its reduced transfer to rice plants after compost application can be linked to the rise in soil organic matter content. Organic matter plays a crucial role in immobilizing Pb by creating stable complexes with it through organic ligands (Turan, 2020). Additionally, with increasing soil pH, Pb^2+^ ions undergo dissociation, leading to the development of negative charges on the substrate's surface functional groups. Consequently, Pb^2+^ ions attach to these surface functional groups, resulting in their removal from the soil solution. This process further reduces Pb bioavailability and prevents its uptake by plants (Sharma and Nagpal, 2018). For context, the Codex Alimentarius Commission sets a maximum level of 0.2 mg/kg Pb for cereal grains; therefore, although grain Pb decreased across treatments, the measured values (0.26–0.44 mg/kg) remain above this benchmark and should be interpreted as a risk-reduction trend rather than full compliance.

### 3.5 Soil enzyme activity

**Figure 4:**
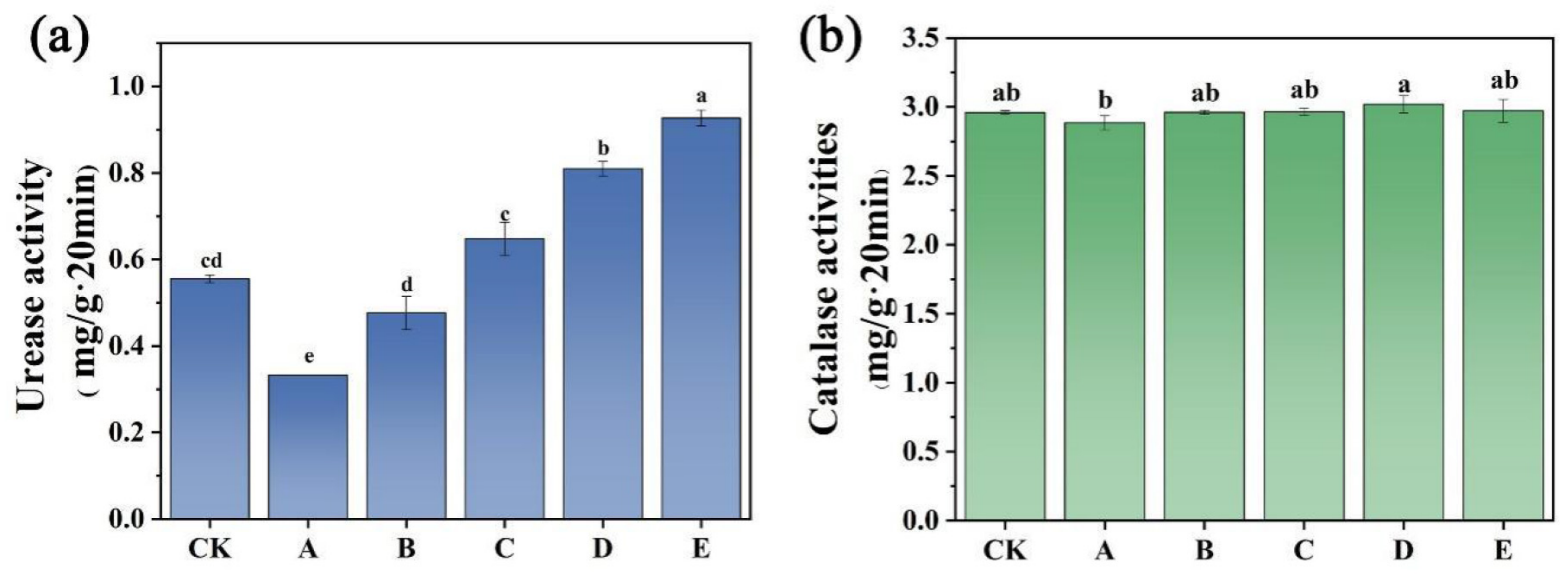
Urease and catalase enzyme activities **(a)** and **(b)** in various soil treatment groups. Error bars represent the standard deviation from three measurements. Different letters denote statistically significant differences between groups at *p* < 0.05. CK (control), A (FMLB), B (FMLB + compost 7:3), C (FMLB + compost 1:1), D (FMLB + compost 3:7), E (compost).

Soil enzymes are vital for biochemical reactions, influencing the direction and strength of these processes, and serving as indicators of soil health (Paz-Ferreiro et al., 2014). Enzyme activities in soil are primarily regulated by the microbial community, which acts as a catalyst for various biochemical reactions. Changes in these biological functions directly reflect enzyme activity, making them potential indicators of ecological risks associated with soil pollutants. Therefore, high concentrations of Pb in soil can disrupt the microbial community, inhibiting soil enzyme activity. Furthermore, enzyme activity serves as an indirect indicator of the soil's ability to self-purify (Yang et al., 2016). Heavy metals can impact enzyme activities by disrupting the microbial community responsible for enzyme synthesis in the soil (Pan and Yu, 2011). In this investigation, catalase and urease activities were measured across various treatment conditions, as depicted in Figure 4.

Catalase, an oxidative reductase enzyme, directly modifies ionic valence and contributes to the detoxification of heavy metals. Amendment addition has been shown to enhance catalase activity in some studies, but no significant effect was observed in this research. Urease is crucial for nitrogen transformation in the soil. Studies indicate that urease activity varies with soil pH and texture, showing greater activity in neutral to mildly acidic conditions compared to alkaline environments (Yang et al., 2016). From Figure 4a, the urease activity observed in Groups A and B (0.33 and 0.47 mg/g·20 min) was notably reduced compared to that in the CK group (0.56 mg/g·20 min), which is likely due to the significant pH increase following the introduction of the materials (Figure 1a). Conversely, the urease activity in Group C (0.65 mg/g·20 min) was 1.16 times greater than in the CK group, likely due to the reduced bioavailability of Pb, which is known to have a significant impact on urease activity in soil (Bååth, 1989). Groups D and E showed urease activity that was 1.45 and 1.66 times greater than that of the CK group, respectively, which can be credited to the substantial organic matter contribution from the compost (Figure 4a). Adding organic matter along with essential nutrients from compost improves soil conditions, stimulating catalase activity, which may enhance soil microbial activity positively (Salam et al., 2019). The catalase levels in soil contaminated with Pb and in soil treated with remediation agents exhibited no significant changes (Figure 4b).

Overall, enzyme activities (urease and catalase) were determined at the end of the pot experiment (day 120) using pooled, air-dried samples to provide a standardized comparison among treatments. We acknowledge that these assays may not fully represent *in situ* enzymatic activities under flooded paddy conditions; therefore, they are interpreted as endpoint indicators of selected microbial functions rather than comprehensive evidence of functional recovery. Future work should incorporate additional enzymes relevant to C, N and P cycling and conduct time-resolved measurements under field-relevant conditions.

### 3.6 Composition of the soil bacterial community

#### 3.6.1 Impact of amendments on bacterial diversity and community structure in contaminated soil

Soil microorganisms are integral to nutrient cycling, and α-diversity serves as an indicator of their ecological function. A higher level of α-diversity contributes to enhanced soil functionality and improved resistance to environmental disturbances (Griffiths and Philippot, 2013). Table 2 presents a comparison of α-diversity indices between original uncontaminated soil (OS) and Pb-treated soil (CK), showing a reduction in both microbial abundance and diversity due to Pb contamination. The amendments applied in this research exhibited a partial restoration of microbial communities. In particular, the introduction of FMLB (Group A) had a marked effect on the microbial composition within Pb-affected soil. Furthermore, applying FMLB and compost in different ratios contributed to the partial restoration of microbial diversity in Pb-impacted soil. The Chao1 and ACE indices, which indicate community abundance, revealed that the values for Group C (FMLB + compost at a 1:1 ratio) were close to those of unpolluted soil, suggesting that this co-remediation strategy partially shifted bacterial diversity toward the OS reference within the pot system.

**Table 2:** Diversity metrics of soil bacterial communities in samples subjected to various amendments.

**Sample**	**Chao1**	**ACE**	**Shannon**
OS	6542.28	7925.08	7.04
CK	6382.50	7144.49	6.78
A	5245.02	5345.17	6.54
B	6303.47	6264.57	6.94
C	6583.50	7815.20	6.72
D	5676.98	5971.34	6.67
E	6023.61	6682.54	6.65

Values are treatment means (n = 3 pots per treatment). OS was maintained under identical pot conditions and included for microbial diversity comparisons. No inferential statistics are shown in this table.

**Figure 5:**
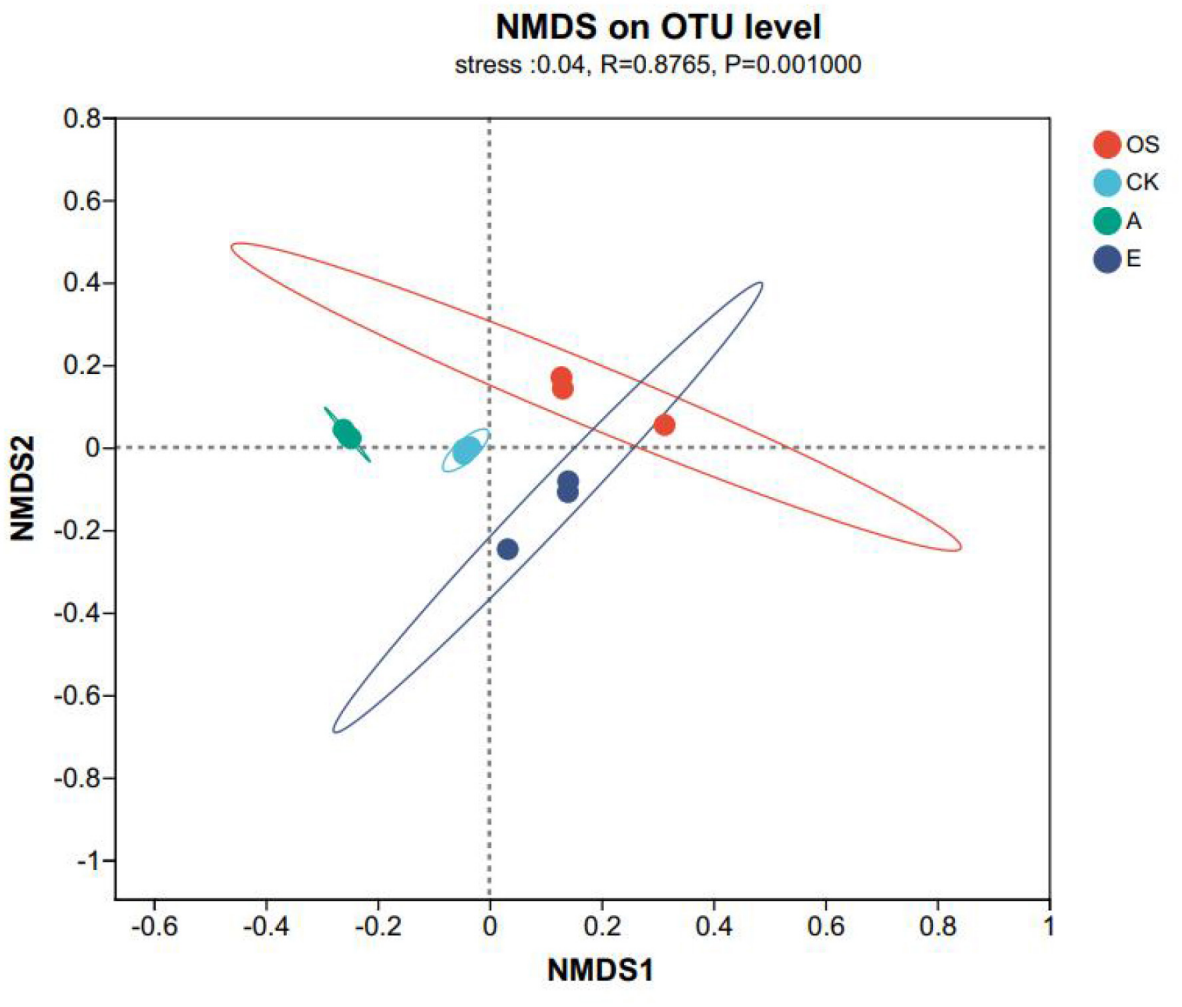
NMDS ordination of Bray–Curtis dissimilarities showing bacterial community structure among treatments (OS: original uncontaminated soil; CK: Pb-contaminated control; A: FMLB; B: FMLB + compost 7:3; C: FMLB + compost 1:1; D: FMLB + compost 3:7; E: compost). PERMANOVA was conducted across all groups, including OS.

The NMDS plot (Figure 5) further shows that soil treated with FMLB and compost had a notably different bacterial community structure compared to both unpolluted soil (OS) and Pb-contaminated soil (*R*^2^ = 0.876, *P* < 0.01). To assess the β-diversity, we conducted a Non-metric Multidimensional Scaling (NMDS) analysis, which visualizes the differences in microbial community composition. The results suggest that the bacterial communities in FMLB-treated soils (Group A) displayed substantial differences from both unpolluted soil (OS) and the Pb-contaminated soil (CK), possibly due to the significant alteration in soil pH caused by the alkaline nature of FMLB. As the proportion of compost increased in Groups D and E, microbial α-diversity gradually decreased, further affecting microbial β-diversity and community structure.

#### 3.6.2 . Impact of soil treatments on bacterial population dynamics and distribution of key taxa

**Figure 6:**
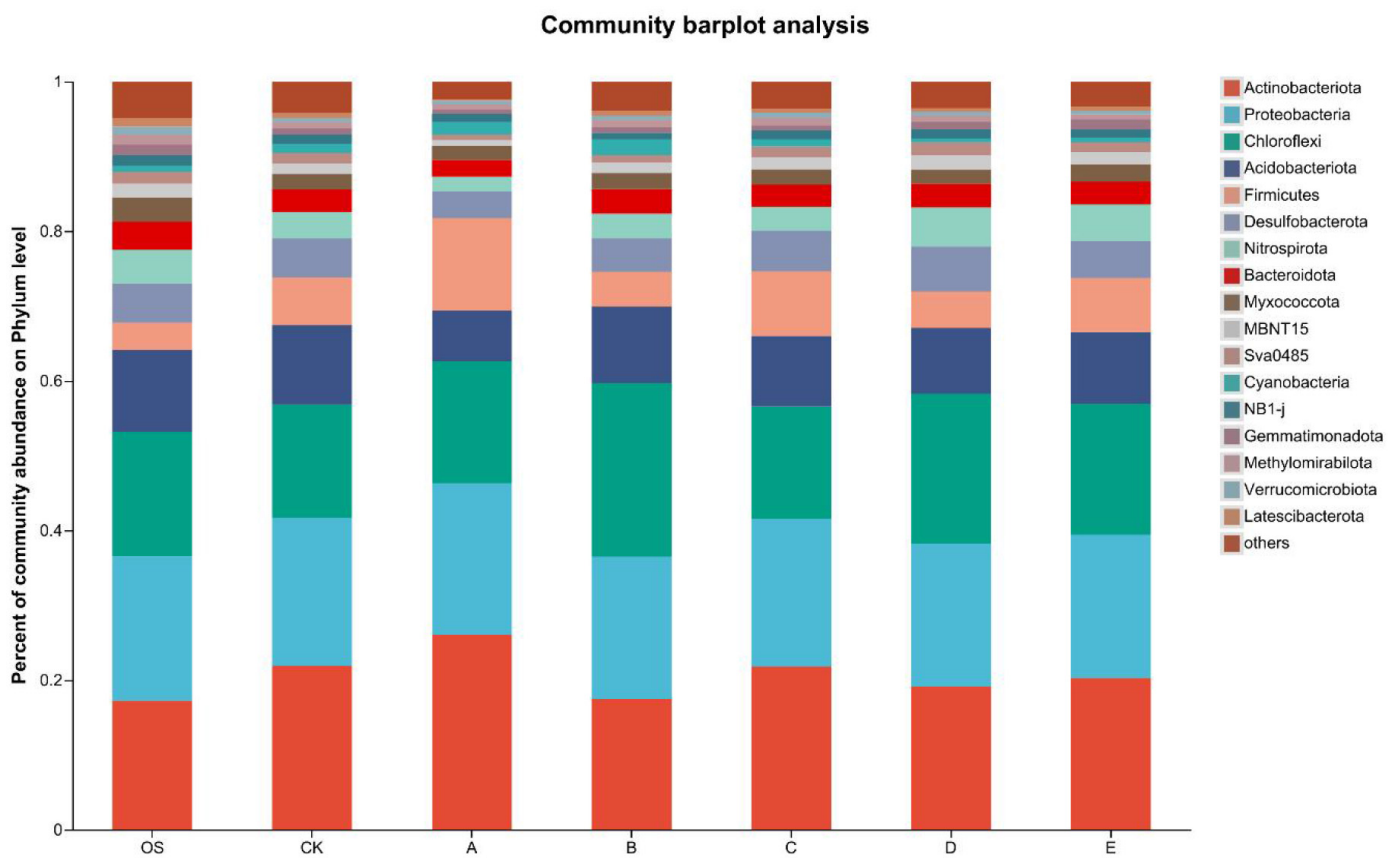
Soil microbial community composition at the Phylum Level across different treatments. OS: original soil; CK (control), A (FMLB), B (FMLB + compost 7:3), C (FMLB + compost 1:1), D (FMLB + compost 3:7), E (compost).

Figure 6 presents the changes in rhizosphere microbial communities in lead-contaminated soil following the application of remediation agents in varying proportions. The bacterial community structure in Pb-affected soil (CK) exhibits notable variations relative to the pristine soil (OS). Groups B, D, and E exhibit microbial communities more similar to OS, whereas the A group treatment led to a distinct shift in microbial community composition. *Actinobacteriota, Proteobacteria, Chloroflexi, Acidobacteriota* and *Firmicutes* were dominant across all treatments. *Proteobacteria* and *Firmicutes* are commonly found in soils contaminated by heavy metals. Additionally, in environments contaminated with heavy metals, phyla like *Proteobacteria, Acidobacteria*, and *Bacteroidota* are typically found in higher abundance. Beyond heavy metal contamination, variables like soil composition, moisture levels, acidity, and nutrient availability—including organic carbon, total nitrogen, and total phosphorus—substantially affect microbial community dynamics (An et al., 2018). Microorganisms such as *Bacteroidota, Proteobacteria*, and *Pontibacter* contribute significantly to the sequestration and stabilization of metallic contaminants. This primarily results from the existence of diverse anionic groups, including peptidoglycan, lipopolysaccharide, and extracellular polysaccharides, on their cell surfaces. These groups help bind heavy metal cations, leading to their immobilization. Additionally, during microbial metabolism, heavy metals may precipitate or weakly bind to macromolecular biopolymers (Tian et al., 2020; Lan et al., 2021).

Kuppusamy et al. reported that *Actinobacteria* predominantly thrive in soils subjected to long-term exposure to pollutants such as polycyclic aromatic compounds and toxic metals, including Pb, Cu, and Zn (Kuppusamy et al., 2016). In comparison to the pristine soil (OS), Pb-contaminated soil (CK treatment) exhibited an increase in *Actinobacteria* proportion, rising from 18.46 % to 21.47 % (Figure 6). The use of remediation agents influenced *Actinobacteria* levels. Incorporating FMLB and compost in different proportions resulted in varying shifts in *Actinobacteria* abundance. Compared to CK, Groups A and C showed an increase, while Groups B, D, and E showed a decrease. In Group E, the relative abundance of *Actinobacteria* decreased to 18.96 %, approaching that of the original uncontaminated soil (OS), while in Group A, the abundance increased to 25.87 %. The presence of FMLB notably influenced *Actinobacteria* populations in the soil, possibly due to the relationship between their abundance and soil pH. The incorporation of FMLB raises soil alkalinity, potentially fostering a higher abundance of *Actinobacteria* (Gao et al., 2021).

Pb contamination significantly altered the abundance of various bacterial phyla in the soil. *Actinobacteria* showed a significant increase in the Pb-contaminated soil (CK), rising from 18.46 % in the original uncontaminated soil (OS) to 21.47 %, suggesting that *Actinobacteria* may play an important role in heavy metal resistance and adaptation to stress (Kuppusamy et al., 2016). In contrast, *Proteobacteria* exhibited a slight decline in abundance, from 20.94 % in OS to 20.46 % in CK. After remediation treatments, Groups A (FMLB alone) and C (FMLB + compost at a 1:1 ratio) showed modest increases in *Proteobacteria* abundance, reaching 21.25 % and 20.7 %, respectively, while Groups B, D, and E showed a decrease, indicating that different remediation strategies had varying effects on this phylum. Notably, Group C exhibited a *Proteobacteria* abundance similar to that of OS, suggesting that the combined application of FMLB and compost may help restore microbial communities to their original state (Chu et al., 2021). These changes reflect the adaptive responses of soil microorganisms to Pb contamination and remediation efforts. The increase in *Actinobacteria* may be related to their resilience in polluted environments, while the decrease in *Proteobacteria* could be linked to the changes in soil conditions after remediation (Azarbad et al., 2015a).

Pb contamination and the application of remediation agents in different proportions both have an impact on the abundance of *Proteobacteria* in the soil, though the effect is not significant. The abundance of *Proteobacteria* in OS was 20.94 %, while in the CK group it was 20.46 %. Groups A and C exhibited a modest rise relative to CK, reaching 21.25 % and 20.7 %, whereas Groups B, D, and E displayed a downward trend, with values of 19.48 %, 18.46 %, and 18.42 %, respectively. Among these, Group C exhibited a *Proteobacteria* abundance more similar to that of OS soil. Earlier research has indicated a connection between *Proteobacteria* and pollutant remediation (Kuppusamy et al., 2016). *Proteobacteria* are the dominant bacterial phylum in heavy metal-contaminated soils and are highly diverse in terms of morphology, physiology, and metabolism, playing a significant role in global carbon, nitrogen, and sulfur cycles (Harichová et al., 2012; Jiang et al., 2019). Due to their unique metabolic capabilities, *Proteobacteria* thrive in heavy metal-contaminated environments and can reduce Pb concentrations by enhancing secondary metabolism.

The *Chloroflexi* phylum is frequently detected in habitats characterized by elevated salinity levels and exposure to toxic metal pollutants. *Chloroflexi* contribute to increasing organic carbon in the soil, promoting microbial respiration, and reducing metal toxicity through processes like external precipitation, enzymatic reactions, surface adsorption, and internal sequestration (Zhang et al., 2020). *Chloroflexi* are anaerobic, and many microorganisms can grow under anaerobic conditions in the rhizosphere. The abundance of *Chloroflexi* varies significantly among different groups. In Pb-contaminated soil (CK), its abundance slightly increased from 14.55 % to 14.66 %. In Group C, the abundance was 14.56 %, which is almost identical to that in the original uncontaminated soil (OS), whereas Groups A, B, D, and E showed abundances of 15.02 %, 22.35 %, 22.09 %, and 20.02 %, respectively, all significantly higher than OS. This variation could be attributed to differences in oxygen levels across the soil samples from various treatment groups.

The *Acidobacteria* phylum is one of the most representative groups in paddy field soils. The abundance of *Acidobacteria* increased after the addition of Pb contamination (CK group), from 9.49 % to 10.56 %. However, in Group A, which received modified materials, there was a significant decrease in abundance, dropping to 6.41 %. Group C showed a slight decrease, while the other groups exhibited an increasing trend. This could be due to changes in the pH of the paddy soil, as soil pH can influence the abundance of *Acidobacteria*, with lower pH values positively affecting its abundance (Shao et al., 2016). Earlier research indicates that *Acidobacteria* tend to thrive in neutral and alkaline conditions, potentially influenced by soil characteristics, while shifts in their abundance might also be linked to the activity of iron ions. Most iron-reducing bacteria belong to the *Acidobacteria* and *Proteobacteria* phyla, and these bacteria may participate in Pb fixation through iron redox reactions. *Acidobacteria* can degrade intricate organic carbon compounds into smaller fatty acids, which may facilitate heavy metal solubilization or absorption (Kulichevskaya et al., 2014).

The abundance of *Firmicutes* increased from 4.34 % to 6.19 % after the addition of Pb contamination (CK group). In Group A, which received FMLB, there was a more significant increase, rising to 12.55%. Groups B and D showed *Firmicutes* abundances of 4.81 % and 4.80 %, respectively, which were similar to the original uncontaminated soil (OS). Studies have indicated that *Firmicutes* often reach higher proportions in environments with severe heavy metal contamination and are frequently found at high-pollution sampling sites (Azarbad et al., 2015b). Additionally, some experiments have observed a potential link between the changes in *Firmicutes* and soil heavy metal stress (Azarbad et al., 2015b). *Firmicutes* are metal-resistant microbes capable of limiting heavy metal availability in the soil. In this research, Pb pollution influenced the microbial assemblage within rice paddies. The application of different remediation agents led to shifts in the microbial community in different directions, all of which were different from the original uncontaminated soil. This may result from the combined impact of alterations in soil properties, Pb contamination, and the use of amended materials. Overall, applying FMLB along with compost in specific ratios (Groups B and D) contributed to aligning the microbial community structure more closely with that of original uncontaminated soil, aiding in minimizing the adverse effects of heavy metal contamination and the influence of remediation materials on soil microbial dynamics (Xu et al., 2016).

**Figure 7:**
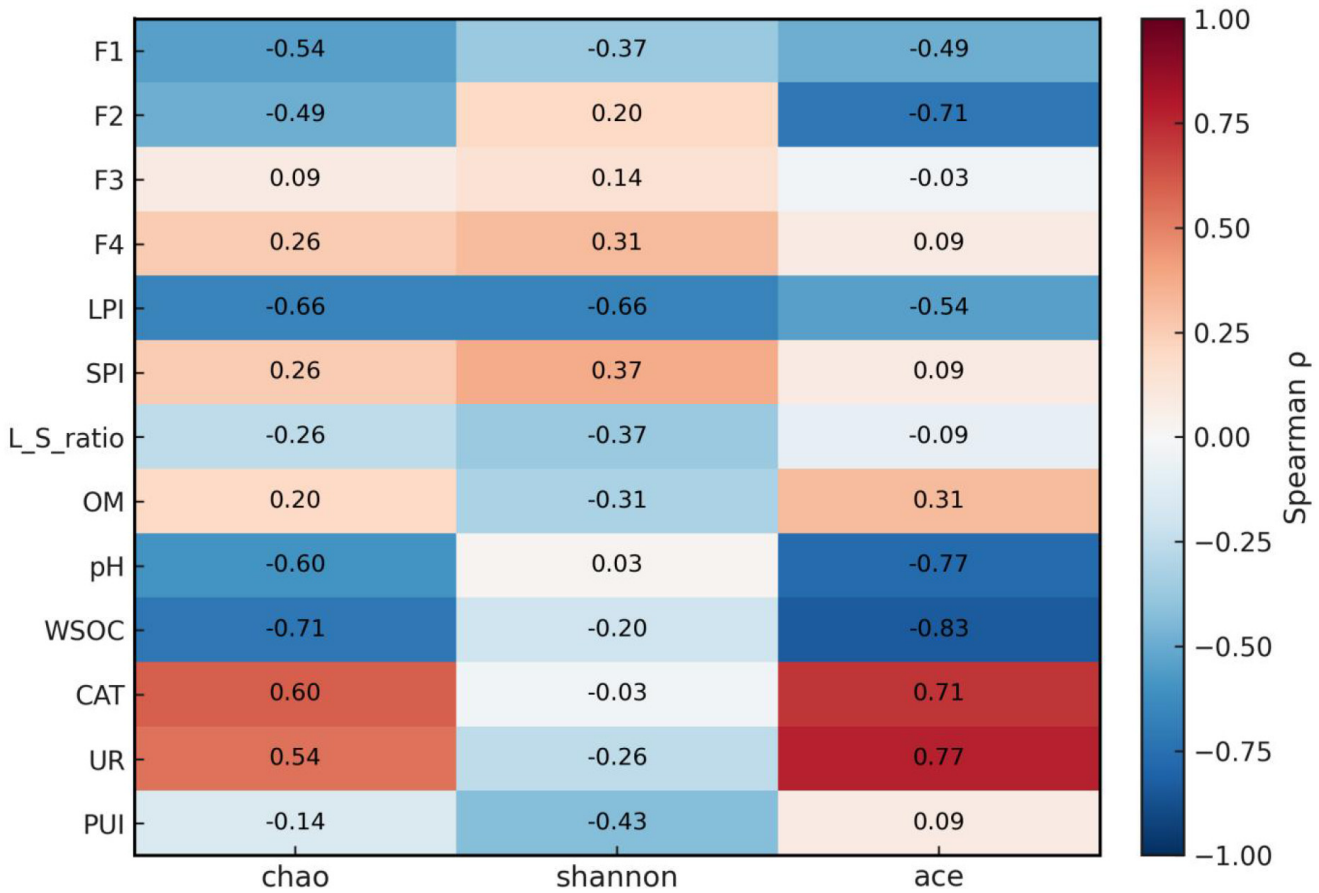
Spearman's rank correlations (ρ) between bacterial α-diversity indices (Chao1, Shannon, ACE) and soil/plant variables in Pb-contaminated paddy soil. Warm colors indicate positive correlations and cool colors indicate negative correlations; numbers in cells are Spearman's ρ. Asterisks denote Benjamini–Hochberg FDR-corrected significance (*p* < 0.05; *p* < 0.01; *p* < 0.001). The heatmap was computed using treatment means of CK and A–E (*n* = 6); therefore, the correlation analysis is exploratory and hypothesis-generating, and causal inference should be avoided, especially given the limited statistical power.

To explore associations between community patterns and soil chemistry/Pb speciation, we computed Spearman correlations across the six treatment means (CK and A–E). Bacterial α-diversity tracked both carbon availability and Pb pools (Figure 7). ACE decreased with WSOC, suggesting that higher labile C favored dominance of a few taxa and reduced estimated richness. By contrast, pH showed no association with Shannon, indicating that the pH range in this study explained little of the diversity pattern. Pb speciation was also informative. The labile Pb index (LPI = F1 + F2) showed negative trends with Chao1 and Shannon, whereas the residual fraction (F4) and the stable pool (SPI = F3 + F4) displayed weak positive associations with diversity. These patterns may be consistent with diversity recovering as bioavailable Pb is converted into more stable forms. Enzyme activities covaried with richness: CAT and UR correlated positively with ACE. In contrast, correlations with a composite plant-uptake index (PUI = z[RE_Pb]+z[RT_Pb]+z[SL_Pb]) were weak, implying that the amendments influenced bacterial communities mainly through soil chemical stabilization rather than via direct plant uptake. Overall, LPI and WSOC showed stronger associations with α-diversity patterns than pH within the studied range. The negative association between ACE and WSOC may reflect that higher labile carbon favors the proliferation of a subset of copiotrophic taxa, potentially reducing estimated richness. Accordingly, compost is discussed here as improving soil habitat quality (e.g., organic matter and nutrient supply) and supporting partial community recovery, rather than uniformly increasing all α-diversity indices. In addition, this correlation analysis is exploratory because it is based on treatment means, and causal inferences should be avoided.

These taxa have been reported to be associated with metal tolerance and immobilization-related processes (e.g., EPS production, organic ligands, and redox-related transformations) in the literature, which has been associated with reduced Pb mobility under certain conditions (Sha et al., 2023). For example, *Actinobacteria* are recognized for their high metal tolerance and production of chelating compounds, while *Acidobacteria* and *Bacteroidota* may assist in Fe–Pb complexation and metal binding (Wang et al., 2019). In previous studies, *Firmicutes*, particularly *Bacillus* species, have been reported to precipitate Pb^2+^ through biomineralization processes (Harpke et al., 2022). However, because this study did not include functional gene assays, metagenomics, or predictive functional profiling, these interpretations should be regarded as literature-supported hypotheses rather than demonstrated mechanisms. Overall, the observed taxonomic shifts are best interpreted as associations with changes in soil chemistry and Pb stabilization.

Beyond geochemical stabilization and plant uptake reduction, this study provides a microbiome-based evaluation framework for organic–inorganic amendment remediation under flooded paddy pot conditions. Specifically, 16S rRNA community profiles (α-diversity and NMDS/PERMANOVA separation) together with endpoint activities of two representative enzymes (urease and catalase) co-varied with shifts in Pb speciation (BCR fractions and LPI/SPI trends), supporting the use of rhizosphere microbiome signatures as sensitive biological indicators associated with Pb stabilization. Importantly, these results are interpreted as treatment-associated community restructuring within the controlled pot system, rather than evidence of microbial-mediated Pb transformation. Overall, several limitations should be acknowledged. This study used a freshly Pb-spiked soil equilibrated for 8 weeks prior to the pot experiment. Because spiked soils may not fully represent long-term aging processes and microbial adaptation in historically contaminated field soils, the observed changes in Pb bioavailability and microbial community metrics should be interpreted as pot-scale responses under controlled conditions, and further validation in field-aged paddy soils is warranted.

## 4 Conclusions

In addition, the coordinated shifts in rhizosphere bacterial community structure and selected enzyme endpoints provide microbiome-associated indicators that complement BCR-based stabilization metrics for evaluating remediation performance under flooded pot conditions. In summary, utilizing FMLB together with compost as a remediation approach proved effective in decreasing Pb bioavailability in polluted paddy soils. The results demonstrate that FMLB has a strong capacity to immobilize Pb through adsorption, ion exchange, and precipitation, which is further enhanced by the addition of compost. The amendments not only lowered Pb levels in the soil but also facilitated the restoration of microbial communities, enhancing overall soil quality and fertility. The most significant reduction in Pb bioavailability and plant uptake was observed when FMLB and compost were applied in a 3:7 ratio, indicating the potential for optimized remediation strategies.

Furthermore, this study highlights the positive impact of integrated organic-inorganic amendments on the soil ecosystem, with particular improvements in microbial diversity and enzyme activity. The findings suggest that using FMLB in combination with compost offers a sustainable, cost-effective, and environmentally friendly approach for remediating Pb-contaminated agricultural soils. These results provide valuable insights for developing effective in situ remediation strategies that ensure food safety and environmental sustainability in heavy metal-contaminated regions.

Correlation analysis further indicated that a decline in the labile Pb index (LPI = F1 + F2; acid-soluble + reducible) together with lower WSOC best explained the partial recovery of bacterial richness, whereas soil pH alone showed little explanatory power within the studied range. Urease and catalase activities were positively aligned with richness, supporting a functional rebound of the microbiome under stabilized Pb conditions. Among all treatments, the FMLB:compost = 3:7 mixture showed the most pronounced trend of redistribution of Pb from more labile fractions (F1–F2) toward less labile fractions (F3–F4; oxidizable + residual) and achieved the lowest grain Pb concentration, supporting a synergistic organic–inorganic remediation effect in which FMLB likely contributes to Pb immobilization while compost moderate alkalization and supports microbial recovery.

## Data Availability

The 16S rRNA amplicon sequencing data have been deposited in the NCBI Sequence Read Archive (SRA) under BioProject PRJNA1405112 (Study accession SRP664492), with Runs SRR36886031 -SRR36886051. All other data supporting the findings of this study are included in the article and its Supplementary material.
