# Supplementary material for: Synergistic remediation of Pb contamination in rice field soil with FeMg-LDH@Bentonite and compost: impacts on Pb bioavailability and soil environment
Source: Front Microbiol. 2026 Feb 6;17:1756444. doi: 10.3389/fmicb.2026.1756444 (PMC12920539; doi:10.3389/fmicb.2026.1756444)
Supplement: Supplementary file 1 [file Supplementary_file_1.docx]

**Synergistic Remediation of Pb Contamination in Rice Field Soil with FeMg-LDH@Bentonite and Compost: Impacts on Pb Bioavailability and Soil Environment^[[1]](#footnote-0)^**

Xian Guan^a^, Jing Bai^b^, Xingzhong Yuan^b^ , Jian-Wei Guo^c^, Xiaowen Liu^a *^.

^a^ Hunan University of Science and Engineering, Yongzhou 425199, PR China

^b^ College of Environmental Science and Engineering, Hunan University, Changsha 410082, P.R. China

^c^ College of Agronomy and Life Sciences, Yunnan Urban Agricultural Engineering and Technological Research Center, Kunming University, Kunming 650214, P.R. China

**Table S1** The community bureau of reference (BCR) extraction method used in this study

| Speciation | Methods |
| --- | --- |
| Acid soluble fraction (F1) | (1) 40 mL 0.11 mol L^-1^ CH_3_COOH (2) shaken at 250 r m^-1^ for 16 h (3) centrifuged at 4000 r m^-1^ for 20 min |
| Reducible fraction (F2) | (1) 40 mL 0.5 mol L^-1^ NH_2_OH·HCl (freshly prepared, pH = 2.0, using 0.5 mol L ^-1^HNO_3_)  (2) shaken at 250 r m^-1^ for 16 h (3) centrifuged at 4000 r m^-1^ for 20 min |
| Oxidizable fraction (F3) | (1) 10 mL H_2_O_2_, kept at 85^◦^C until the mixture volume was less than 3 mL (2) 10 mL H_2_O_2_, kept at 85^◦^C until the mixture volume was less than 1 mL (3) 50 mL NH_4_OAC (pH = 2.0, using HNO_3_) (4) shaken at 250 r m^-1^ for 16 h (5) centrifuged at 4000 r m^-1^ for 20 min |
| Residual fraction (F4) | (1) a mixture of 5 mL HNO_3_, 5 mL HF and 3 mL HClO_4_ (2) 90^◦^C for 40 min, 140^◦^C for 60 min, 170^◦^C for 40 min (3) dilute the products with ultrapure water to a volume of 100 mL |


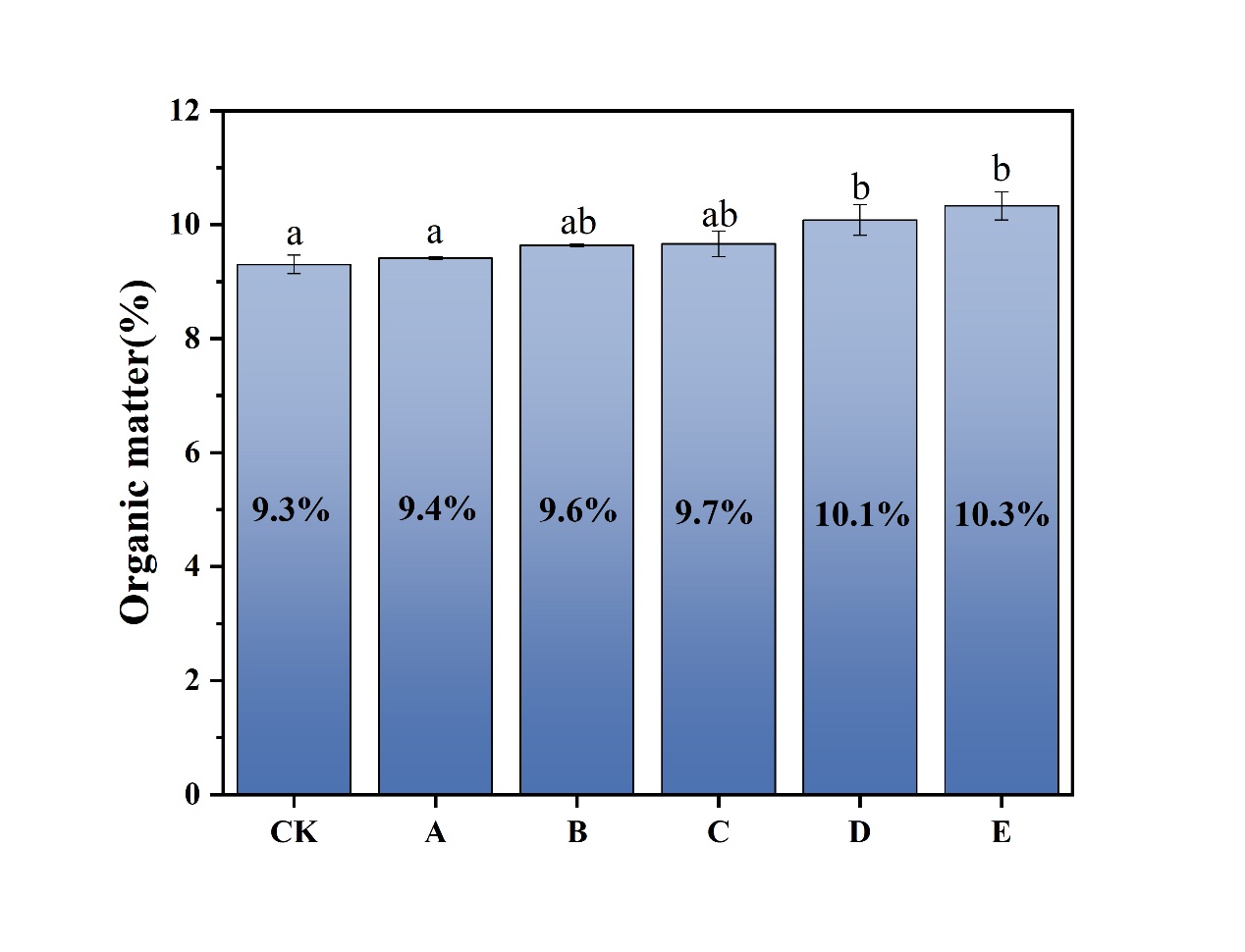


**Fig. S1.** Influence of different amendments on soil organic matter (OM). Error bars represent the standard deviation from three measurements. Different letters denote statistically significant differences between groups at *p* < 0.05. The treatments were as follows: CK (control), A (FeMg-LDH@Bentonite), B (FeMg-LDH@Bentonite + compost at a 7:3 ratio), C (FeMg-LDH@Bentonite + compost at a 1:1 ratio), D (FeMg-LDH@Bentonite + compost at a 3:7 ratio), and E (compost).

1. ^*^Corresponding author at: Hunan University of Science and Engineering, Yongzhou 425199, PR China; E-mail address:liuxiaowen@huse.edu.cn (XW.Liu). [↑](#footnote-ref-0)
